# TCR repertoire divergence reflects micro-environmental immune phenotypes in glioma

**DOI:** 10.1186/2051-1426-2-S3-O19

**Published:** 2014-11-06

**Authors:** Jennifer Sims, Boris Grinshpun, Yaping Feng, Justin Neira, Jorge Samanamud, Peter Canoll, Peter Sims, Yufeng Shen, Jeffrey Bruce

**Affiliations:** 1 Columbia University, New York, NY, USA; 2 Rutgers University, New Brunswick, NJ, USA

## Background & significance

Glioblastoma (GBM) remains prognostically dismal, with only modest gains in mean survival time with chemo- and radiotherapy motivating research into reversing its characteristic local and systemic immunosuppression with precision in this high-risk tissue. While whole-repertoire amplification of the TCR repertoire allows unprecedented depth regarding the potentiation of anti-tumor responses, most studies utilize TCRseq for monitoring reactivity to specific tumor antigens, or the identities of particular TCRs as biomarkers. In this study, we have utilized whole-repertoire analysis to describe the relationship between intra-tumoral T cells and peripheral circulation, and leverage mutual information between gene expression and the behavior of the T cell population to characterize glioma-reactive states, driven by the gene expression of the principal resident monocyte population, and perturbable by immunological interventions.

## Methods & results

From resected tumor tissue and peripheral lymphocytes of low- and high-grade human glioma patients, TCRseq libraries were generated using reverse transcription and nested PCR (iRepertoire [1]) of the complementarity-determining region 3 (CDR3) of the TCR-alpha and TCR-beta chains, then sequenced on an Illumina MiSeq. We developed a computational pipeline for mapping TCR cassettes, *in silico *translation, and sequence error correction from these libraries, enabling sensitive calculation of tumor-infiltrating lymphocyte (TIL) and peripheral TCR diversity (Shannon entropy) [2], as well as the divergence (Jensen-Shannon divergence metric) between the two T cell populations.

By integrating amino acid identity and V-J cassette combination, we observed varying levels of divergence between the TIL and peripheral lymphocytes of glioma patients, and changes in this divergence over tumor progression in a PDGF-driven murine model. Correlation of these properties with tumor tissue RNA profiling, by differential gene expression and mutual-information gene ontology, revealed an association between tumor growth and high blood-brain TCR divergence - particularly in amino-acid sequence, suggesting antigen-driven selection - while high expression of inflammatory and certain immune pathway markers computationally attributed to microglia [3] were anti-correlated with divergence. Preliminary murine experiments suggest that TCR divergence can be altered by induction and blockade of cytokine-mediated activation of these pathways.

## Conclusion

The expression of a subset of microglia-associated genes appears to describe micro-environmental states which are strongly tied to the tumor-specificity of the intra-tumoral TCR repertoire, complementary to the tumor-centric classifications of TCGA. TCRseq-based profiling not only promises to inform tailoring of local and systemic immunotherapy to target the most relevant immunosuppressive mechanisms, but may also provide non-invasive assessment of the intra-tumoral environment for refined diagnosis and monitoring during clinical trials.
